# Experimental infection of pigs with H1 and H3 influenza A viruses of swine by using intranasal nebulization

**DOI:** 10.1186/s12917-018-1434-z

**Published:** 2018-03-27

**Authors:** Nobuhiro Takemae, Ryota Tsunekuni, Yuko Uchida, Toshihiro Ito, Takehiko Saito

**Affiliations:** 1 Division of Transboundary Animal Disease, National Institute of Animal Health, NARO, 3-1-5 Kannondai, Tsukuba, Ibaraki 305-0856 Japan; 2 Thailand–Japan Zoonotic Diseases Collaboration Center, Kasetklang, Chatuchak, Bangkok 10900 Thailand; 3 The Avian Zoonosis Research Center, Faculty of Agriculture, Tottori University, 4-101 Koyama-cho Minami, Tottori, Tottori 680-8550 Japan; 4 United Graduate School of Veterinary Sciences, Gifu University, 1-1, Yanagito, Gifu, Gifu 501-1112 Japan

**Keywords:** Influenza, Pig, Nebulization, Clinical sign, Viral shedding, Infection, Inoculation, Experimental

## Abstract

**Background:**

Experimental infection of pigs via direct intranasal or intratracheal inoculation has been mainly used to study the infectious process of influenza A viruses of swine (IAVs-S). Nebulization is known to be an alternative method for inoculating pigs with IAVs-S, because larger quantities of virus potentially can be delivered throughout the respiratory tract. However, there is very little data on the experimental infection of pigs by inhalation using nebulizer. In the current study, we used intranasal nebulization to inoculate pigs with 9 different IAVs-S—3 H1N1, 2 H1N2, and 4 H3N2 strains. We then assessed the process of infection by evaluating the clinical signs, nasal and oral viral shedding, and seroconversion rates of the pigs inoculated.

**Results:**

Lethargy and sneezing were the predominant clinical signs among pigs inoculated with 7 of the 9 strains evaluated; the remaining 2 strains (1 H1N1 and 1 H1N2 isolate) failed to induce any clinical signs throughout the experiments. Significantly increased rectal temperatures were observed with an H1N1 or H3N2 strains between 1 and 3 days post-inoculation (dpi). In addition, patterns of nasal viral shedding differed among the strains: nasal viral shedding began on 1 dpi for 6 strains, with all 9 viruses being shed from 2 to 5 dpi. The detection of viral shedding was less sensitive from oral samples than nasal secretions. Viral shedding was not detected in either nasal or oral swabs after 10 dpi. According to hemagglutination–inhibition assays, all inoculated pigs had seroconverted to the inoculating virus by 14 dpi, with titers ranging from 10 to 320.

**Conclusions:**

Our current findings show that intranasal nebulization successfully established IAV-S infections in pigs and demonstrate that clinical signs, viral shedding, and host immune responses varied among the strains inoculated.

## Background

Swine influenza, due to influenza A viruses of swine (IAVs-S), is a component of the porcine respiratory disease complex, which causes substantial economic losses in the pig industry. Swine influenza in pig farms typically is characterized by acute respiratory disease accompanied by fever, nasal discharge, cough, anorexia, and lethargy [1]. IAVs-S are spread among pigs through the nasopharyngeal route or by aerosols that are generated naturally through coughing and exhalation [2, 3]. Clinical signs quickly spread throughout piggeries or pig farms, and affected animals usually recover rapidly from illness [1]. In addition to the typical epizootic form showing clinical signs, IAVs-S have frequently been recovered from pigs without clinical signs, especially in the weaning and early-fattening stages [4–7]. Regardless of the severity, IAVs-S infection has adverse effects on the growth performance of pigs on farms [8].

Many experimental infection studies have been conducted to elucidate the infectious process of IAVs-S in pigs [9–15]. Pigs experimentally infected develop similar clinical signs as seen under natural infection [2], but different inoculation methods cause differences in pathogenicity, even among the same strain. Typically direct intranasal and intratracheal inoculation methods have been used to establish experimental infections in pigs. Direct intranasal inoculation, in which the inoculum is delivered into the nasal cavity itself by using a syringe or mucosal atomization device, is the easiest option, but it is associated with variable efficacy in establishing infections, because pigs may swallow most of the inoculum [2]. In contrast, intratracheal inoculation is believed to increase the reliability and reproducibility of the course of infection in lung [16], but nasal shedding of virus sometimes is decreased in both titer and duration compared with direct intranasal inoculation of the same strain [17]. Specifically, in the cited study, the duration of nasal virus shedding in the pigs inoculated intranasally with the avian-like IAV-S A/swine/Belgium/1/1998 (H1N1) was 5 days, whereas that in pigs inoculated intratracheally with the same strain was 3 days [17]. In addition, no respiratory disease symptoms occurred in pigs inoculated intranasally with A/swine/Ghent/172/2008 (H3N2), but swine inoculated intratracheally with the same strain developed prominent coughing, sneezing, and nasal discharge [18]. Some researchers have adopted the mixed inoculation method [14, 19, 20]. For example, Romagosa et al., infected the pigs intratracheally and intranasally with a total of 2 ml of the H1N1 IAV-S at a titer of 1 × 10^6^ TCID_50_/ml [19].

Nebulization that may enable the delivery of large quantities of IAVs-S throughout the respiratory tract is expected to be used for experimental infection with IAVs-S in pigs [2]. However, the application of nebulization for experimental inoculation of IAVs-S in pigs is limited [21–23], perhaps because this method is more labor-intensive than other methods. In the current study, we used intranasal nebulization to infect pigs with several IAVs-S and then assessed the infection process by measuring nasal and oral viral shedding, determining seroconversion, and monitoring the clinical signs of infected pigs.

## Methods

### Influenza A viruses

To assess inoculation by the intranasal nebulization method, nine field strains of IAVs-S (Table 1) representing 7 genotypes were used and comprised two pandemic A(H1N1)2009 viruses (A(H1N1)pdm09v) [A/swine/Yamagata/11/2010(H1N1) (Yam10) and A/swine/Kagoshima/30/2015(H1N1) (Kag15)], a classical H1N2 IAVs-S [A/swine/Tochigi/1/2008(H1N2) (Toc08)], two H1 reassortants between classical H1 IAVs-S and A(H1N1)pdm09v [A/swine/Tochigi/1/2012(H1N2) (Toc12) and A/swine/Chiba/1-11/2015(H1N1) (Chi15)], a seasonal human-origin H3N2 IAVs-S [A/swine/Nagano/2000(H3N2) (Nag00)], an H3 reassortant between seasonal human-like H3N2 and A(H1N1)pdm09v (A/swine/Miyazaki/2/2013(H3N2) (Miy13)], and two North American Triple reassortant (TR) IAVs-S [A/swine/Yokohama/aq114/2011(H3N2) (Yok11) and A/swine/Minnesota/01146/2006(H3N2) (Min06)] (Table 1).

**Table 1:** Gene constellation of influenza A viruses of swine used in this study

Virus	Abbreviation	Subtype	H1 clade^c^	Genetic origin								Isolate ID or accession no.^d^
				HA	NA	PB2	PB1	PA	NP	M	NS	
A/swine/Yamagata/11/2010^a^	Yam10	H1N1	1A.3.3.2	Pdm	Pdm	Pdm	Pdm	Pdm	Pdm	Pdm	Pdm	EPI_ISL_237800
A/swine/Kagoshima/30/2015^a^	Kag15	H1N1	1A.3.3.2	Pdm	Pdm	Pdm	Pdm	Pdm	Pdm	Pdm	Pdm	EPI_ISL_237923
A/swine/Chiba/1-11/2015^a^	Chi15	H1N1	1A.1	Cla	Pdm	Pdm	Pdm	Pdm	Cla	Pdm	Pdm	EPI_ISL_221941
A/swine/Tochigi/1/2008^a^	Toc08	H1N2	1A.1-like	Cla	Hu	Cla	Cla	Cla	Cla	Cla	Cla	EPI_ISL_237796
A/swine/Tochigi/1/2012^a^	Toc12	H1N2	1A.1-like	Cla	Hu	Pdm	Pdm	Pdm	Pdm	Pdm	Pdm	EPI_ISL_237798
A/swine/Nagano/2000^a^	Nag00	H3N2	N/A	Hu	Hu	Hu	Hu	Hu	Hu	Hu	Hu	EPI_ISL_237795
A/swine/Miyazaki/2/2013^a^	Miy13	H3N2	N/A	Hu	Hu	Pdm	Pdm	Pdm	Pdm	Pdm	Pdm	EPI_ISL_237794
A/swine/Yokohama/aq114/2011	Yok11	H3N2	N/A	TR	TR	TR	TR	TR	TR	TR	TR	AB741020-AB741027
A/swine/Minnesota/01146/2006^b^	Min06	H3N2	N/A	TR	TR	TR	TR	TR	TR	TR	TR	CY099035-CY099042

*Cla* classical swine lineage, *Hu* seasonal human-like lineage, *TR* North American triple-reassortant lineage

^a^Complete genomic sequence was obtained during this study

^b^Kindly provided by Dr. Amy L Vincent (US Department of Agriculture, Ames, IA, USA)

^c^H1 clade was obtained by Swine H1 Clade Classification Tool [https://www.fludb.org/]. *N/A* not applicable

^d^Isolate IDs from GISAID; accession numbers from GenBank

Four of these field strains (Yam10, Toc08, Toc12, and Miy13) were isolated from domestic pigs showing apparent clinical disease, such as sneezing, at pig farms. Strains Chi15 and Kag15 were isolated from domestic pigs without apparent clinical disease at pig farms. Nag00 that was isolated from a pig at a slaughterhouse was kindly supplied by the Nagano Environmental Conservation Research Institute, Japan. Yok11 that was isolated from a clinically healthy pig under quarantine at Animal Quarantine Office, Japan (Kanagawa, Yokohama, Japan) was submitted to the National Institute of Animal Health, Japan, for diagnosis. The other TR cluster IV strain, Min06 (kindly provided by Dr. Amy L. Vincent, US Department of Agriculture, Ames, IA, USA), was used for comparison with Japanese strains. All of the strains were isolated and grown by using MDCK cells before inoculation.

The complete genomic sequences of all isolates except for Yok11 and Min06 were obtained during the current study by using next-generation sequencing (Miseq, Illumina, San Diego, CA, USA) or Sanger sequencing (ABI model 3130, Applied Biosystems, Foster City, CA, USA) as previously described [24]. The complete genomic sequences of Yok11 were analyzed in our previous study [25] and those of Min06 had been already deposited in GenBank [https://www.ncbi.nlm.nih.gov/genbank/].

### Animals and inoculation by nebulization

The study population comprised 40 specific pathogen-free (Large White × Landrace) crossbred pigs (obtained at 4 weeks of age; Zen-noh Premium Pig, Zen-Noh, Ibaraki, Japan) that were confirmed to be serologically negative for influenza A viruses (Influenza Ab Test Kit, IDEXX Laboratories, Westbrook, ME, USA) at 1 week prior to inoculation and were acclimated to Biosafety Level 3 housing (National Institute of Animal Health, Tsukuba, Ibaraki, Japan) for at least 5 days prior to inoculation. Four pigs were housed in each isolation unit (235 [w] × 365 [d] × 290 [h] cm; AIRTEC JAPAN, Tokyo, Japan), which were equipped with HEPA filters and passive airflow, throughout the experiment. Pigs in groups inoculated with Yam10, Toc08, or Toc12 were co-housed without partitions in each isolation unit, while all other pigs (including controls) were individually housed behind stainless-steel dividers in each isolation unit because the experimental rooms were renovated to assess the effect of IAV-S infection in individual pigs more accurately.

**Fig. 1:**
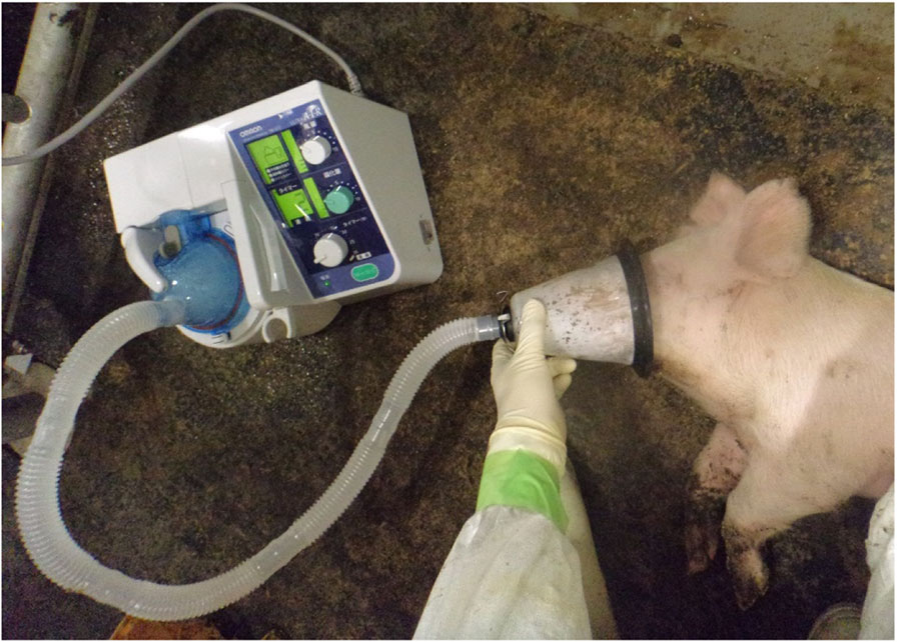
Intranasal nebulization to inoculate pigs with IAVs-S by using the nebulizer unit

Just prior to inoculation, pigs (age, 5 weeks) were anesthetized with medetomidine (50 μg/kg IM; Domitor, Zenoaq, Fukushima, Japan) and midazolam (500 μg/kg IM; Dormicum, Astellas Pharma, Tokyo, Japan). Each strain was administered at a dose of 2 × 10^6^ TCID_50_/ml to 4 pigs by using a nebulizer unit (NE-U17, OMRON, Kyoto, Japan) with a fitted nose cone (Fig. 1). The device that is capable of generating aerosolized particles (diameter, 1 to 8 μm) by ultrasonic waves delivered the virus-containing particles at 0.5 ml/min for 4 min. After viral inoculation, anesthesia was reversed by using atipamezole (5.0 mg/mL; Antisedan, Zenoaq). In addition, four control pigs were inoculated with sterile PBS in the same manner.

### Clinical monitoring and sample collection

All of the pigs examined were observed every morning for about 30 min for clinical signs of disease, such as sneezing, lethargy, and diarrhea until 14 dpi. The sneezing episode was defined either as a single sneeze or continuous sneezes. The lethargy was defined based on the response of each pig towards the investigators. The diarrhea was apparent when the rectal temperatures of the pigs were measured. The anorexia was defined by whether the pigs took the food or not. The pigs were scored for the clinical signs as follows: sneezing [S] 0 – absent, 1 – present; lethargy [L] 0 – absent, 1 – present; diarrhea [D] 0 – absent, 1 – present. All scores per pig group are accumulated for a total clinical score of each individual pig. The score of anorexia was not accumulated because Yam10, Toc08, or Toc12 pig groups were co-housed without partitions in each isolation unit, which made unable to identify which pigs had anorexia.

Rectal temperatures of the pigs infected with Kag15, Chi15, Nag00, Miy13, Yok11, and Min06 and of the 4 control pigs were measured in the morning at 0, 1, 2, 3, 4, 5, 7, 10, and 14 dpi by using a thermometer (Thermo flex, Measure Technology, Taiwan). We were unable to obtain accurate rectal temperatures from the pigs inoculated with Yam10, Toc08, or Toc12 because these pigs were able to move around freely before temperature assessment.

To evaluate viral shedding, nasal swab samples were collected individually from all pigs at 1, 2, 3, 4, 5, 7, 10, and 14 days post-infection (dpi) by using flocked swabs with a plastic handle (Ex Swab 001, Denka Seiken, Tokyo, Japan) and placed in 2 ml of medium (MEM containing penicillin [1000 unit/ml], streptomycin [1000 μg/ml], and Fungizone [25 μg/ml]; Thermo Fisher Scientific, Waltham, MA, USA], 0.01 M HEPES, and 0.5% bovine serum albumin); samples were centrifuged at 1400×*g* for 5 min, and supernatant was collected, aliquoted, and stored at − 80 °C until use.

In addition, oral swabs were collected at 1, 3, 5, 7, 10, and 14 dpi from the pigs infected with Kag15, Chi15, Yok11, Nag00, Miy13, and Min06, and rectal swabs were collected at 1, 2, 3, 4, 5, 7, 10, and 14 dpi from the pigs infected with Yam10, Toc08, and Toc12. All of these samples were processed, aliquoted, and stored in the same manner as the nasal swab samples.

At 14 dpi, all pigs were anesthetized by using ketamine (30 mg/kg IM; Ketalar, Daiichi-Sankyo, Tokyo, Japan) and euthanized by exsanguination. Then the lungs were removed and evaluated macroscopically.

### Analysis of viral shedding

To remove any bacterial contaminants before use, supernatants from nasal, oral, and rectal swabs were thawed, vortexed, and filtered through polyvinylidene difluoride membrane, which has low protein-binding ability (pore size, 0.45 μm; catalog no. SLHVJ13SL, Millipore, Billerica, MA, USA). Each sample was diluted ten-fold serially into serum-free MEM supplemented with 1 μg/ml TPCK-treated trypsin (Thermo Fisher Scientific) and antibiotics; 25 μl of each dilution was inoculated onto PBS-washed confluent MDCK cells in 96-well plates. After 5 days of incubation at 37 °C under 5% CO_2_, the TCID_50_ of each sample was calculated according to hemagglutination activity by using 0.55% red blood cells from guinea pigs.

### Serologic assays

Blood samples were collected at 3 days before inoculation and at 14 dpi for use in ELISA and hemagglutination-inhibition (HAI or HI) assays. Sera were evaluated by ELISA (Influenza Ab Test Kit, IDEXX Laboratories) according to the manufacturer’s instructions. To remove nonspecific hemagglutinin inhibitors from the samples before their use in the HAI assay, sera were treated with a 20% suspension of kaolin (Sigma-Aldrich, St. Louis, MO, USA) after heat inactivation at 56 °C for 30 min and then adsorbed to 0.55% guinea pig red blood cells for 20 min at room temperature. HAI assays were conducted according to the WHO manual on Animal Influenza Diagnosis and Surveillance [26].

### Statistical analyses

Data were analyzed by using RStudio Version 1.0.136 [27]. In all analyses, the level of significance was *P* < 0.05.

## Results

### Clinical signs

Inoculation of IAVs-S by nebulization led to mild clinical signs in each group, except for those exposed to Yam10 or Toc08 (Table 2). The high clinical scores ranging from 13 to 18 were observed in the Miy13, Yok11, Kag15, and Chi15 groups. All pigs from these groups demonstrated at least one clinical signs assessed. The Min06, Toc12, and Nag00 groups showed middle clinical scores ranging from 4 to 10. Two or three of the four pigs showed at least one clinical sign in these groups. Lethargy and sneezing were the most common signs among the clinical signs observed and occurred between 3 and 12 dpi in 18 and 15, respectively, of the 36 pigs inoculated with IAVs-S (Table 2). These clinical signs occurred most frequently in the Miy13 and Yok11 groups. Specifically, all pigs inoculated with Miy13 were lethargic and sneezing by 9 dpi, and 3 of the 4 pigs in the Yok11 group were similarly affected by 8 dpi (Table 2).

**Table 2:** Clinical signs and clinical scores of each pig or pig group after inoculation with IAV-S by nebulization

Infected with	PigID	Day after infection												Total clinical score
		3	4	5	6	7	8	9	10	11	12	13	14	
Yam10	1													0
	2													
	3													
	4													
Kag15	1	S, L		S		S, L					S			13
	2	L												
	3	S, L			L	L								
	4	L				L								
Chi15	1				L		L							13
	2	S		L	L		L							
	3			L	L		L							
	4			L	L	L	L							
Toc08	1													0
	2													
	3													
	4													
Toc12	1													7
	2	S												
	3												D	
	4					D	D	D		D			D	
Nag00	1			L	S	S		L						10
	2							L						
	3	S	S	S										
	4					S	S							
Miy13	1	S	S, L		L	S	S, L	S						18
	2		L				S, L							
	3				S		S, L							
	4			S			S, L	L						
Yok11	1			L	S				A	A	A			14
	2		S	L		L								
	3			L		L	L							
	4		S	L	S	L	S, L							
Min06	1													4
	2													
	3		S	S										
	4	S					S							

*A* anorexia, *L* Lethargy, *S* sneezing, *D* diarrhea

^a^Data for days 1 and 2 are not shown because none of the pigs in any group showed any clinical signs evaluated in this study

In addition, the clinical signs differed markedly between Yam10 and Kag15, although both are A(H1N1)pdm09 viruses (Table 1). Whereas all 4 pigs inoculated with Kag15 became lethargic between 3 and 7 dpi, those inoculated with Yam10 were clinically healthy throughout the experiments (Table 2). Overall, although clinical signs were most prevalent between 3 and 8 dpi, two pigs inoculated with Toc12 had diarrhea between 7 and 14 dpi (Table 2). In addition, one pig inoculated with Yok11 had anorexia from 10 to 12 dpi (Table 2).

**Fig. 2:**
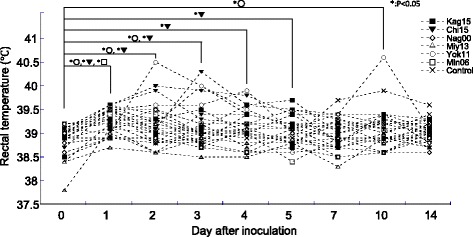
Rectal temperatures of pigs from 0 to 14 days after inoculation. Pig groups in which the average body temperature was significantly (*P* < 0.05, Dunnet’s test) higher than normal (38.9 ± 0.31 °C; calculated by using 0-dpi data from all pigs tested) are shown by asterisks

We measured the body temperature of the pigs inoculated with Kag15, Chi15, Nag00, Miy13, Yok11, or Min06 and the control pigs. The normal body temperature (mean ± 1 SD on 0 dpi) of these 28 pigs was 38.9 ± 0.31 °C. Average rectal temperatures in 3 groups—those inoculated with Chi15, a reassortant H1 classical IAVs-S, and the 2 H3 TR IAVs-S, Yok11 and Min06—were higher than normal by 5 dpi (*P* < 0.05, Dunnet’s test; Fig. 2). Specifically, 2 of the 4 pigs inoculated with Chi15 were febrile (that is, rectal temperature greater than 40 °C) at 2 and 3 dpi, as were 2 pigs in the Yok11 group. In addition, one of the pigs in the Yok11 group that had an increased temperature at 3 dpi later developed anorexia and a fever of 40.6 °C at 10 dpi (Table 2). In the Min06 group, the pigs’ average rectal temperature was increased (39.4 °C ± 0.17) only at 1 dpi (*P* < 0.05, Dunnet’s test). None of the groups inoculated with the A(H1N1)pdm09v Kag15 or the 2 human-like H3 IAVs-S, Nag00 and Miy13, developed a significantly increased rectal temperature throughout the experiment.

### Viral shedding and macroscopic lesions

**Fig. 3:**
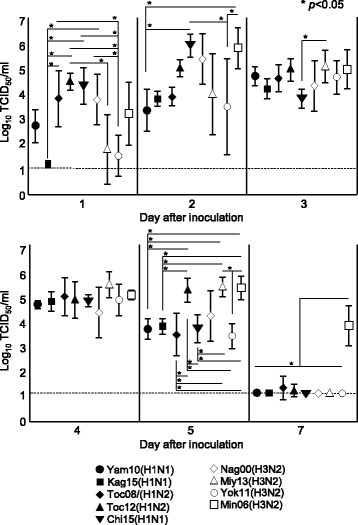
Viral titers (mean ± 1 standard deviation) from nasal swabs of pigs inoculated with various IAVs-S by nebulization. No virus was shed from the control pigs throughout the experiment or from the inoculated pigs after 10 dpi. *, Significant difference (*P* < 0.05, Tukey HSD test) between groups on the same day; the dashed line indicates the limit of detection (10^1.1^TCID_50_/ml)

Inoculation of IAVs-S by inhalation using nebulizer unit evoked nasal viral shedding in all of the nine pig groups and the titer of each strain reached similar levels at titers of 10^4.3^ to 10^5.4^ TCID_50_/ml at 4 dpi (Fig. 3). On the other hand, samples from the nasal swabs, except for those collected at 4 dpi, revealed different patterns of viral shedding among the evaluated strains (Fig. 3). Pigs typically began shedding virus at 1 dpi, but 2 pigs each in the Kag15 and Yok11 groups and 3 pigs in the Miy13 group did not shed any virus at 1 dpi. However all inoculated pigs shed virus at titers of 10^1.2^ to 10^6.9^ TCID_50_/ml from 2 to 5 dpi. By 7 dpi, all the pigs except for 1 pig each in the Toc08 and Toc12 groups and 4 pigs in Min06 group had stopped shedding virus. No virus was shed from the inoculated pigs after 10 dpi.

According to nasal swab samples, the average virus titer did not differ between the groups inoculated with Yam10 and Kag15, the 2 A(H1N1)pdm09v strains, throughout the experiment. However, unlike those in the Kag15 group, all of the pigs in Yam10 group shed virus, at an average titer of 10^2.7^ TCID_50_/ml, at 1 dpi (Fig. 3). The titers of the A(H1N1)pdm09 viruses were numerically lower than those of other classical H1 and H3N2 IAVs-S. The average titers of the Kag15 group were lower than those of pigs inoculated with Toc08, Toc12, Chi15, or Nag00 at 1 dpi and lower than those of the Toc12, Miy13, and Min06 groups at 5 dpi (*P* < 0.05, Tukey HSD test) (Fig. 3). In addition, Yam10 titers were lower than those of Chi15 and Min06 at 2 dpi and lower than those of Toc12, Miy13, and Min06 at 5 dpi (*P* < 0.05, Tukey HSD test) (Fig. 3). Among the groups inoculated with classical H1 virus, the titer of Toc12 peaked at 10^5.2^ TCID_50_/ml at 5 dpi and was higher than the titers of Toc08 and Chi15 on the same day (*P* < 0.05, Tukey HSD test) (Fig. 3). No significant differences in virus titers occurred between the human-like H3 IAVs-S Nag00 and Miy13. Between the TR IAVs-S, the average titers of the Min06 group at 2, 5, and 7 dpi were higher than those of the Yok11 group (*P* < 0.05, Tukey HSD test) (Fig. 3). In addition, Min06 replicated the most efficiently among the IAVs-S tested; the average Min06 titer of 10^3.8^ TCID_50_/ml at 7 dpi was higher than those of all other groups (*P* < 0.05, Tukey HSD test) (Fig. 3).

**Fig. 4:**
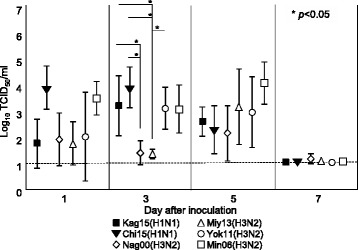
Viral titers (mean ± 1 standard deviation) from oral swabs of pigs inoculated with Kag15, Chi15, Nag00, Miy13, Yok11, and Min06 by nebulization. No virus was shed from the inoculated pigs after 10 dpi. *, Significant difference (*P* < 0.05, Tukey HSD test) between groups on the same day; dashed line indicates the limit of detection (10^1.1^TCID_50_/ml)

We also collected oral fluids from the pigs inoculated with Kag15, Chi15, Yok11, Nag00, Miy13, or Min06 to compare the viral shedding data between oral and nasal swab samples (Fig. 4). Viral titers determined from oral swabs were significantly lower than those from nasal swabs in all 6 groups at either 3 or 5 dpi. The average oral virus titers at 5 dpi were 10^2.6^ TCID_50_/ml for Kag15, 10^2.3^ TCID_50_/ml for Chi15, 10^4.1^ TCID_50_/ml for Min 06, 10^2.2^ TCID_50_/ml for Nag00, and 10^3.2^ TCID_50_/ml for Miy13 and were lower than the nasal swab titers of 10^3.8^ TCID_50_/ml, 10^3.7^ TCID_50_/ml, 10^5.3^ TCID_50_/ml, 10^4.2^ TCID_50_/ml, and 10^5.3^ TCID_50_/ml, respectively, at the same time point (*P* < 0.05; t-test). Yok11 yielded an oral virus titer of 10^3.1^ TCID_50_/ml at 3 dpi, which was lower than the nasal virus titer with (10^4.6^ TCID_50_/ml) on the same day (*P* < 0.05, t-test). In addition, Nag00 yielded oral virus titers of 10^1.9^ TCID_50_/ml at 1 dpi and 10^1.4^ TCID_50_/ml at 3 dpi, which were significantly lower than the nasal virus titers with (10^3.8^ TCID_50_/ml and 10^4.3^ TCID_50_/ml) on the same day (*P* < 0.05, t-test). Among the 6 groups, the average titers of the Nag00 and Miy13 groups were significantly lower than those of pigs inoculated with Kag15 or Chi15 at 3 dpi (*P* < 0.05, Tukey HSD test) (Fig. 4). The average titer of the Miy13 group was also significantly lower than that of pigs inoculated with Yok11 at 3 dpi (*P* < 0.05, Tukey HSD test) (Fig. 4). No virus was detected in the oral fluid of all the inoculated pigs after 10 dpi. Rectal swabs from pigs inoculated with Yam10, Toc08, or Toc12 failed to yield any virus throughout the study.

At 14 dpi, we removed the lungs from all pigs for macroscopic evaluation. Pneumonia-like lesions were present in one pig inoculated with Toc08. These lesions were purple to dark-red, consolidated areas in the apical parts of the cranial, middle, and caudal lobes (data not shown). In contrast, none of the other pigs (infected or control) had any macroscopic lung lesions at 14 dpi.

### Serologic assays

By 14 dpi, all of the 36 inoculated pigs had seroconverted, according to results of HAI assays using each challenge strain as the antigen (Table 3). In contrast, the mean sample: negative ratios of the ELISAs at 14 dpi tended to be lower than the values before virus inoculation, such that 23 of the 36 virus-inoculated pigs were considered to be seronegative at 14 dpi (Table 3).

**Table 3:** Serologic analyses of sera obtained 3 days before (Pre) and 14 days post-infection (dpi)

Virus	Pig ID	HAI titer^a^		S:N of ELISA^b^			
		Pre	14 dpi	Pre		14 dpi	
Yam10(H1N1)	1	< 10	80	0.95	–	0.72	–
	2	< 10	80	0.86	–	0.88	–
	3	< 10	80	0.97	–	0.68	–
	4	< 10	80	0.88	–	0.85	–
Kag15(H1N1)	1	< 10	20	0.96	–	0.73	–
	2	< 10	160	0.89	–	0.31	+
	3	< 10	160	0.91	–	0.61	–
	4	< 10	80	0.93	–	0.53	+
Toc08(H1N2)	1	< 10	20	1.04	–	0.76	–
	2	< 10	20	1.02	–	0.62	–
	3	< 10	10	0.91	–	0.66	–
	4	< 10	10	1.06	–	0.66	–
Toc12(H1N2)	1	< 10	80	0.85	–	0.71	–
	2	< 10	80	0.78	–	0.56	+
	3	< 10	160	0.96	–	0.39	+
	4	< 10	80	0.90	–	0.52	+
Chi15(H1N1)	1	< 10	160	1.08	–	0.59	+
	2	< 10	80	0.91	–	0.56	+
	3	< 10	160	0.87	–	0.55	+
	4	< 10	80	0.86	–	0.56	+
Nag00(H3N2)	1	< 10	160	0.94	–	0.79	–
	2	< 10	80	0.71	–	0.74	–
	3	< 10	80	0.70	–	0.72	–
	4	< 10	160	0.84	–	0.44	+
Miy13(H3N2)	1	< 10	320	0.72	–	0.78	–
	2	< 10	320	1.02	–	0.78	–
	3	< 10	160	0.70	–	0.59	+
	4	< 10	160	0.73	–	0.72	–
Yok11(H3N2)	1	< 10	320	0.93	–	0.74	–
	2	< 10	80	0.93	–	0.70	–
	3	< 10	160	0.95	–	0.62	–
	4	< 10	160	0.82	–	0.47	+
Min06(H3N2)	1	< 10	320	1.00	–	0.72	–
	2	< 10	320	0.68	–	0.96	–
	3	< 10	160	0.93	–	0.96	–
	4	< 10	80	0.75	–	0.49	+
Control	1	< 10	< 10	0.84	–	0.95	–
	2	< 10	< 10	0.82	–	0.93	–
	3	< 10	< 10	0.82	–	0.95	–
	4	< 10	< 10	0.86	–	0.95	–

^a^Homologous viruses were used as antigens in each group. Sera from control pigs were tested against classical H1 (Chi15), A(H1N1)pdm09v (Kag15), and triple-reassortant (Yok11) IAVs-S as antigens

^b^For swine sera, a sample:negative (S:N) ratio < 0.6 was considered positive (+; −, negative), according to the manufacturer’s instructions

The kinetics of the immune responses differed among the IAVs-S we evaluated. For example, among the groups inoculated with classical H1 strains, the immune responses in pigs against Toc08 were relatively lower than those against the reassortant classical H1 IAVs-S (Toc12 and Chi15). Specifically, the geometric mean HAI titers at 14 dpi were 14, 95, and 113 in the Toc08, Toc12, and Chi15 groups, respectively. In addition, all of the pigs inoculated with Toc08 were considered seronegative according to ELISA, but at least 3 pigs each in the Toc12 and Chi15 groups were seropositive. In addition, whereas all of the H3N2-inoculated pigs had HAI titers greater than 80, only one pig in each of these groups was considered seropositive according to ELISA (Table 3), suggesting no clear correlation between HAI titers and ELISA sample: negative values at 14 dpi.

## Discussion

We demonstrated that nebulization successfully evoked viral shedding from nasal cavities and seroconversion in all of the pigs inoculated with any of the IAVs-S evaluated in this study. As might have been expected [2], the intranasal nebulization method was highly effective for delivering the viruses to the upper respiratory tracts of pigs. In fact, in humans and ferrets, aerosolized influenza viruses efficiently reach both the upper respiratory tract as well as deep into the lower regions [28, 29]. The nebulizer we used in the current study produces aerosolized particles that are 1 to 8 μm in diameter. Whereas aerosol particles larger than 6 μm are trapped preferentially in the upper respiratory tract, those smaller than 5 μm are efficiently deposited in the lower respiratory tract [28]. In one study, nebulization of swine A(H1N1)pdm09v successfully infected pigs at a much lower dose than did direct intranasal administration [22]. Together these previous findings and our current results support the idea that the nebulization method can deliver sufficient virus to establish IAV-S infection in pigs.

The severity of clinical signs and duration of virus shedding in pigs appears to vary not only according to the IAV-S inoculated but also according to protocols used. In our present study, the severity of the clinical signs in pigs infected through nebulization differed among the different strains of IAVs-S. For example, Chi15 induced lethargy more often than did the other H1 IAVs-S, Toc08 and Toc12. In addition, among H3N2 IAVs-S, Yok11 and Min06, but not Nag00 or Miy13, caused pigs to become febrile. Recent studies have revealed differences in virulence among IAVs-S strains isolated after 2009. For example, 6 different TR H3N2 IAVs-S that resulted from repeated spillover of A(H1N1)pdm09v from humans to pigs demonstrated distinct patterns of viral shedding and different degrees of pathology in experimentally infected pigs [14]. In addition, disease characteristics, such as percentage of pneumonia and viral titer in lungs, reportedly differed significantly among H1 strains from the United States [12]. In the cited study, H1 IAVs-S isolated after 1999 tended to produce more severe disease and greater viral shedding in pigs than did classical strains, such as A/swine/Iowa/15/1930 (H1N1).

In our current study, results from the HAI test and ELISA assay were discordant in some cases. The Influenza Ab Test Kit (Idexx Laboratories) detects only the antibodies produced against a highly conserved epitope of the influenza A nucleoprotein (NP) that binds virus RNA [30]. In contrast, HAI tests detect anti-HA antibodies, which predominate in the sera of animals infected with influenza A viruses. In general, HAI antibodies can be detected in the sera of pigs by 7 dpi and peak 2 to 3 weeks after primary infection [31, 32]. However, NP-specific IgG antibody responses after primary infection with IAVs-S arise later than HAI antibodies in pig serum. For example, the ELISA titers of pigs inoculated with 10^8^ TCID_50_/ml of avian-like H1N1 or H3N2 IAVs-S peaked 1 month after infection [33]. Together these data support our results in which ELISA titers were undetectable at 14 dpi in pigs that were shown to be infected in light of viral shedding.

Oral fluids collected from pigs by hanging a cotton rope in the pen have been proposed to be a useful tool for the detection of porcine circovirus 2 and porcine reproductive and respiratory syndrome virus in the context of increasing the cost-effectiveness and efficiency of sampling for surveillance of swine herds [34, 35]. However, for IAVs-S, our results indicate that virus titers from oral swabs are lower than those from nasal swabs, even when collected from the same pigs at the same time point by using flocked swabs. Similarly, other experiments have shown that the virus isolation rate from pigs experimentally inoculated with IAVs-S is lower from oral swabs than nasal swabs [36, 37]. However those studies relied on real-time RT-PCR analysis rather than virus isolation, suggesting that oral fluids might effectively be used for the detection of IAVs-S genes. In another study, viral RNA in pigs experimentally infected with A/swine/Gent/28/2010 (H1N1) was detectable in oral fluids at 21 dpi but in nasal swabs at 5 dpi [38]. Therefore, virus detection using pen-based oral fluid samples might decrease the cost and number of samples per farm compared with nasal swab collection from individual pigs. However, great care needs to be exercised during the genetic characterization of IAVs-S obtained from pen-based oral fluid samples, because whether the detected IAVs-S are reassortant viruses or multiple viruses derived from several pigs cannot be determined. That genetically different IAVs-S circulate concurrently within a single farm is unsurprising [6, 24, 39].

## Conclusions

In conclusion, regardless of the strain, intranasal nebulization of IAVs-S successfully established infections in all of the inoculated pigs. The nebulization method is already well established for the experimental infection of horses with influenza A viruses [40, 41]. Our current study demonstrates that nebulization can be useful for the experimental infection of pigs as well. The application of this method to pigs has the potential to advance our understanding of the progression of IAVs-S infections within and between pigs and to facilitate vaccine efficacy tests in swine.

## Abbreviations

A(H1N1)pdm09v: Pandemic A(H1N1)2009 virus
dpi: Days post-inoculation
IAV-S: Influenza A virus of swine
TCID_50_: 50% of tissue infectious dose

## References

[1] Olsen CW, Brown IH, Easterday BC, Van Reeth K, Straw DJ, Zimmerman JJ, d'Alaaire S, Taylor DJ (2006). Swine influenza. Diseases of swine.

[2] Janke BH, Richt JA, Webby RJ (2013). Clinicopathological Features of Swine Influenza. Swine Influenza.

[3] Alonso C, Raynor PC, Davies PR, Torremorell M (2015). Concentration, Size Distribution, and Infectivity of Airborne Particles Carrying Swine Viruses. PLoS One.

[4] Takemae N, Shobugawa Y, Nguyen PT, Nguyen T, Nguyen TN, To TL (2016). Effect of herd size on subclinical infection of swine in Vietnam with influenza A viruses. BMC Vet Res.

[5] Ozawa M, Matsuu A, Yonezawa K, Igarashi M, Okuya K, Kawabata T (2015). Efficient isolation of swine influenza viruses by age-targeted specimen collection. J Clin Microbiol.

[6] Corzo CA, Culhane M, Juleen K, Stigger-Rosser E, Ducatez MF, Webby RJ (2013). Active surveillance for influenza A virus among swine, Midwestern United States, 2009–2011. Emerg Infect Dis.

[7] Takemae N, Parchariyanon S, Ruttanapumma R, Hiromoto Y, Hayashi T, Uchida Y (2011). Swine influenza virus infection in different age groups of pigs in farrow-to-finish farms in Thailand. Virol J.

[8] Er C, Lium B, Tavornpanich S, Hofmo PO, Forberg H, Hauge AG (2014). Adverse effects of Influenza A(H1N1)pdm09 virus infection on growth performance of Norwegian pigs - a longitudinal study at a boar testing station. BMC Vet Res.

[9] Landolt GA, Karasin AI, Phillips L, Olsen CW (2003). Comparison of the Pathogenesis of Two Genetically Different H3N2 Influenza A Viruses in Pigs. J Clin Microbiol.

[10] Richt JA, Lager KM, Janke BH, Woods RD, Webster RG, Webby RJ (2003). Pathogenic and antigenic properties of phylogenetically distinct reassortant H3N2 swine influenza viruses cocirculating in the United States. J Clin Microbiol.

[11] Choi Y, Goyal S, Joo H (2004). Evaluation of transmission of swine influenza type A subtype H1N2 virus in seropositive pigs. Am J Vet Res.

[12] Vincent AL, Lager KM, Ma W, Lekcharoensuk P, Gramer MR, Loiacono C (2006). Evaluation of hemagglutinin subtype 1 swine influenza viruses from the United States. Vet Microbiol.

[13] Kitikoon P, Nilubol D, Erickson BJ, Janke BH, Hoover TC, Sornsen SA (2006). The immune response and maternal antibody interference to a heterologous H1N1 swine influenza virus infection following vaccination. Vet Immunol Immunopathol.

[14] Rajão DS, Walia RR, Campbell B, Gauger PC, Janas-Martindale A, Killian ML (2017). Reassortment between Swine H3N2 and 2009 Pandemic H1N1 in the United States Resulted in Influenza A Viruses with Diverse Genetic Constellations with Variable Virulence in Pigs. J Virol.

[15] Ma W, Vincent AL, Lager KM, Janke BH, Henry SC, Rowland RRR (2009). Identification and characterization of a highly virulent triple reassortant H1N1 swine influenza virus in the United States. Virus Genes.

[16] Winkler GC, Cheville NF (1986). Ultrastructural morphometric investigation of early lesions in the pulmonary alveolar region of pigs during experimental swine influenza infection. Am J Pathol.

[17] De Vleeschauwer A, Atanasova K, Van Borm S, van den Berg T, Rasmussen TB, Uttenthal Å (2009). Comparative Pathogenesis of an Avian H5N2 and a Swine H1N1 Influenza Virus in Pigs. PLoS One.

[18] Pomorska-Mol M, Kwit K, Markowska-Daniel I, Kowalski C, Pejsak Z (2014). Local and systemic immune response in pigs during subclinical and clinical swine influenza infection. Res Vet Sci.

[19] Romagosa A, Allerson M, Gramer M, Joo H, Deen J, Detmer S (2011). Vaccination of influenza a virus decreases transmission rates in pigs. Vet Res.

[20] Kitikoon P, Vincent A, Gauger P, Schlink S, Bayles D, Gramer M (2012). Pathogenicity and transmission in pigs of the novel A(H3N2)v influenza virus isolated from humans and characterization of swine H3N2 viruses isolated in 2010-2011. J Virol.

[21] Thacker EL, Thacker BJ, Janke BH (2001). Interaction between Mycoplasma hyopneumoniae and Swine Influenza Virus. J Clin Microbiol.

[22] Hemmink JD, Morgan SB, Aramouni M, Everett H, Salguero FJ, Canini L (2016). Distinct immune responses and virus shedding in pigs following aerosol, intra-nasal and contact infection with pandemic swine influenza A virus, A(H1N1)09. Vet Res.

[23] Swenson SL, Vincent LL, Lute BM, Janke BH, Lechtenberg KE, Landgraf JG (2001). A Comparison of Diagnostic Assays for the Detection of Type a Swine Influenza Virus from Nasal Swabs and Lungs. J Vet Diagn Invest.

[24] Takemae N, Harada M, Nguyen PT, Nguyen T, Nguyen TN, Tol TL, et al. Influenza A Viruses of Swine (IAV-S) in Vietnam from 2010 to 2015: Multiple Introductions of A(H1N1)pdm09 Viruses into the Pig Population and Diversifying Genetic Constellations of Enzootic IAV-S. J Virol. 2017;91(1): e01490–16. 10.1128/JVI.01490-16 PMC5165217 27795418

[25] Matsuu A, Uchida Y, Takemae N, MawatariI T, Yoneyama S, Kasai T (2012). Genetic characterization of swine influenza viruses isolated in Japan between 2009 and 2012. Microbiol Immunol.

[26] WHO.WHO Manual on Animal Influenza Diagnosis and Surveillance. In:WHO Global Influenza Programme. World Health Organization. 2002. http://www.who.int/csr/resources/publications/influenza/en/whocdscsrncs20025rev.pdf. Accessed 07 Apr 2017.

[27] RStudio-Team.RStudio. In:Integrated Development for R. RStudio, Inc. 2015. http://www.rstudio.com/. Accessed 18 Apr 2017.

[28] Raymond T (2006). Review of Aerosol Transmission of Influenza A Virus. Emerg Infect Dis.

[29] Gustin KM, Belser JA, Wadford DA, Pearce MB, Katz JM, Tumpey TM (2011). Influenza virus aerosol exposure and analytical system for ferrets. Proc Natl Acad Sci U S A.

[30] IDEXX-Laboratories.Influenza A Ab Test Information Sheet. In:IDEXX Laboratories, Inc. 2010. https://www.idexx.com/en/livestock/livestock-tests/swine-tests/idexx-swine-influenza-virus-ab-test/. Accessed 22 Mar 2018.

[31] Larsen DL, Karasin A, Zuckermann F, Olsen CW (2000). Systemic and mucosal immune responses to H1N1 influenza virus infection in pigs. Vet Microbiol.

[32] Van Reeth K, Labarque G, Pensaert M (2006). Serological profiles after consecutive experimental infections of pigs with European H1N1, H3N2, and H1N2 swine influenza viruses. Viral Immunol.

[33] Heinen P, De Boer-Luijtze E, Bianchi T (2001). Respiratory and systemic humoral and cellular immune responses of pigs to a heterosubtypic influenza A virus infection. J Gen Virol.

[34] Ramirez A, Wang C, Prickett JR, Pogranichniy R, Yoon K-J, Main R (2012). Efficient surveillance of pig populations using oral fluids. Prev Vet Med.

[35] De Regge N, Cay B (2016). Comparison of PRRSV Nucleic Acid and Antibody Detection in Pen-Based Oral Fluid and Individual Serum Samples in Three Different Age Categories of Post-Weaning Pigs from Endemically Infected Farms. PLoS One.

[36] Detmer SE, Patnayak DP, Jiang Y, Gramer MR, Goyal SM (2011). Detection of Influenza a Virus in Porcine Oral Fluid Samples. J Vet Diagn Invest.

[37] Goodell CK, Prickett J, Kittawornrat A, Zhou F, Rauh R, Nelson W (2013). Probability of detecting influenza A virus subtypes H1N1 and H3N2 in individual pig nasal swabs and pen-based oral fluid specimens over time. Vet Microbiol.

[38] Decorte I, Steensels M, Lambrecht B, Cay AB, De Regge N (2015). Detection and Isolation of Swine Influenza A Virus in Spiked Oral Fluid and Samples from Individually Housed, Experimentally Infected Pigs: Potential Role of Porcine Oral Fluid in Active Influenza A Virus Surveillance in Swine. PLoS One.

[39] Abe H, Mine J, Parchariyanon S, Takemae N, Boonpornprasert P, Ubonyaem N (2015). Co-infection of influenza A viruses of swine contributes to effective shuffling of gene segments in a naturally reared pig. Virology.

[40] Mumford JA, Hannant D, Jessett DM (1990). Experimental infection of ponies with equine influenza (H3N8) viruses by intranasal inoculation or exposure to aerosols. Equine Vet J.

[41] Yamanaka T, Nemoto M, Bannai H, Tsujimura K, Kondo T, Matsumura T (2016). Assessment of antigenic difference of equine influenza virus strains by challenge study in horses. Influenza Other Respi Viruses.

